# Unraveling the Mechanism of Cooperative Redox Chemistry in High‐Efficient Zn^2+^ Storage of Vanadium Oxide Cathode

**DOI:** 10.1002/advs.202305749

**Published:** 2023-11-14

**Authors:** Lijun Zhou, Ping Li, Chenghui Zeng, Ang Yi, Jinhao Xie, Fuxin Wang, Dezhou Zheng, Qi Liu, Xihong Lu

**Affiliations:** ^1^ School of Applied Physics and Materials Wuyi University Jiangmen 529020 P. R. China; ^2^ College of Chemistry and Chemical Engineering Research Center for Ultra Fine Powder Materials Key Laboratory of Functional Small Organic Molecule Ministry of Education and Jiangxi's Key Laboratory of Green Chemistry Jiangxi Normal University Nanchang 330022 P. R. China; ^3^ MOE of the Key Laboratory of Bioinorganic and Synthetic Chemistry The Key Lab of Low‐carbon Chem & Energy Conservation of Guangdong Province School of Chemistry Sun Yat‐Sen University Guangzhou 510275 P. R. China; ^4^ Department of Physics City University of Hong Kong Hong Kong 999077 P. R. China

**Keywords:** cooperative redox chemistry, electron carrier, high capacity, vanadium oxide, Zn ion batteries

## Abstract

The inferior capacity and cyclic durability of V_2_O_5_ caused by inadequate active sites and sluggish kinetics are the main problems to encumber the widespread industrial applications of vanadium‐zinc batteries (VZBs). Herein, a cooperative redox chemistry (CRC) as “electron carrier” is proposed to facilitate the electron‐transfer by capturing/providing electrons for the redox of V_2_O_5_. The increased oxygen vacancies in V_2_O_5_ provoked in situ by CRC offers numerous Zn^2+^ storage sites and ion‐diffusion paths and reduces the electrostatic interactions between vanadium‐based cathode and intercalated Zn^2+^, which enhance Zn^2+^ storage capability and structural stability. The feasibility of this strategy is fully verified by some CRCs. Noticeably, VZB with [Fe(CN)_6_]^3−^/[Fe(CN)_6_]^4−^ as CRC displays conspicuous specific capacity (433.3 mAh g^−1^), ≈100% coulombic efficiency and superb cyclability (≈3500 cycles without capacity attenuation). Also, the mechanism and selection criteria of CRC are specifically unraveled in this work, which provides insightful perspectives for the development of high‐efficiency energy‐storage devices.

## Graphical abstract

An effective strategy by introducing suitable cooperative redox chemistry (CRC) is proposed to enhance electrochemical performance of V_2_O_5_. Attributed to the characteristic of capturing/providing electrons for V_2_O_5_ redox and the in situ increased oxygen vacancy in V_2_O_5_, the Zn battery with V_2_O_5_ (V_2_O_5_//Zn) shows conspicuous capacity and cyclability. Also, the underlying mechanism and selection criteria of CRC are elucidated in depth.

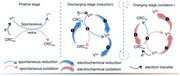

## 1 Introduction

The actualization of carbon neutrality is promoted by developing the long‐lasting, sustainable but intermittent energies, whose integration and utilization reply primarily on the energy storage systems.^[^ ^1^ ^]^ It is acknowledged that lithium‐ion batteries with the virtues of high energy density and reversible performance have occupied the predominant energy market as yet.^[^ ^2^ ^]^ Nevertheless, the shortage of lithium resources and safety issues from combustible organic electrolytes (involving fire disaster and explosion hazard) considerably retard the large‐scale utilization of Li‐ion batteries.^[^ ^3^ ^]^ In comparison, Zn‐ion batteries have drawn increasing attention in view of their environmental compatibility, non‐inflammability, suitable redox potential (−0.76 V versus the SHE electrode in a weakly acidic condition) and high theoretical capacities (820 mAh g^−1^ and 5851 mAh cm^−3^).^[^ ^4^ ^]^ Over the last few years, multiple cathode materials have been extensively developed to match with the high‐capacity Zn metal anode, mainly including manganese‐based compounds,^[^ ^5^ ^]^ vanadium‐based materials,^[^ ^6^ ^]^ Prussian blue analogues,^[^ ^7^ ^]^ metal sulphides^[^ ^8^ ^]^ and organic polymers.^[^ ^9^ ^]^ Thereinto, V_2_O_5_ featuring cost‐effectiveness (≈US $7.85/1b), environmental friendliness, multivalency of vanadium and high theoretical capacity of 589 mAh g^−1^ (based on the reversible V^5+^/V^4+^ and V^4+^/V^3+^ two‐electron transfer reaction) are deemed as a representative cathode material for Zn‐based batteries.^[^ ^6b^ ^]^ However, the low capacity and poor cyclic durability resulting from the deficient Zn^2+^ storage sites and depressed kinetics of V_2_O_5_ cathode hinder further commercialization of vanadium‐zinc batteries (VZBs).^[^ ^10^ ^]^

Until now, some mature strategies have been dedicated to enhance the electrochemical properties of V_2_O_5_. Among them, pre‐intercalation engineering is one of most prevailing strategies to optimize V_2_O_5_ structure. For example, Yang and co‐workers pre‐intercalated H_2_O into the vanadium oxides to weaken the electrostatic interactions between Zn^2+^ and V_2_O_5_ framework, thus exhibiting a markedly improved cycling stability with 71% capacity retention after 900 cycles.^[^ ^11^ ^]^ Moreover, an interface modification strategy was also capitalized on constructing V_2_O_5_@poly(3,4‐ethylenedioxythiophene) (V_2_O_5_ by Xu et al., which is favorable for improving the capacity (360 mAh g^−1^) and cycling durability (89% capacity retention after 1000 cycles) of V_2_O_5_‐based electrode.^[^ ^12^ ^]^ Additionally, Zhang and co‐workers endowed the V_2_O_5_‐based Zn‐ion battery with great stability (>82% capacity retention after 800 cycles) by introducing rich oxygen vacancy in V_2_O_5_.^[^ ^13^ ^]^ Electrolyte regulation has also been validated to be an effective strategy for optimizing the electrochemical performance of V_2_O_5_. For instant, a Zn(CF_3_SO_3_)_2_ based “Water‐in‐Salt” electrolyte with an extremely high concentration of 21 m LiTFSI additive enabled the Zn/V_2_O_5_ battery with a high discharge capacity of 238 mAh g^−1^ and enhanced lifespan (80% of the initial discharge capacity after 2000 cycles).^[^ ^14^ ^]^ Notwithstanding the considerable development achieved, the overall performance of V_2_O_5_ cathode is still far from the expectations for practical applications, considering the high‐cost and limited ion conductivity of a super concentrated electrolyte, harsh conditions for defect generation, and time‐consuming structure optimization. Thereupon, it is challenging but urgently needed to exploit a facile and available approach to achieve high‐efficiency V_2_O_5_ cathodes.

In this work, we propose a straightforward but effective strategy to enhance the electrochemical performance of VZBs by introducing a suitable cooperative redox chemistry (CRC) as “electron carrier”. First, the characteristic of spontaneous reaction with V_2_O_5_ electrode enables the selected CRC to facilitate electron transport by capturing/providing electrons for the V^5+^/V^4+^ and V^4+^/V^3+^ redox. Second, the in situ increased oxygen vacancy in V_2_O_5_ stimulated by reductive CRC (denoted as CRC_re_) is anticipated to provide more Zn^2+^ storage sites and ion‐diffusion paths and weaken the interaction between Zn^2+^ and the V_2_O_5_ host material, thus improving Zn^2+^ storage capability, kinetics, and structural stability of V_2_O_5_. Such a strategy is substantiated by the CRCs including [Fe(CN)_6_]^3−^/[Fe(CN)_6_]^4−^ (FeCN‐CRC) and I_3_^−^/I^−^ (I‐CRC), which not only possess the characteristics of spontaneous reaction with V_2_O_5_ and reversible electrochemical redox reaction the potential window of VZBs, but also cause no additional side effects in VZBs system. As a proof of concept demonstration, Zn battery with FeCN‐CRC additive in electrolyte manifests a specific capacity of 433.3 mAh g^−1^ at 0.1 A g^−1^, together with a maximum energy density of 338.6 Wh kg^−1^ and peak power density of 3332.9 W kg^−1^. A superior cycling stability is also obtained over 3500 cycles without obvious capacity fading. Moreover, the underlying mechanism and selection criteria of CRC in vanadium oxide cathode revealed in this work open up new opportunities to achieve high‐efficiency energy‐storage devices.

## 2 Results and Discussion

**Figure 1:**
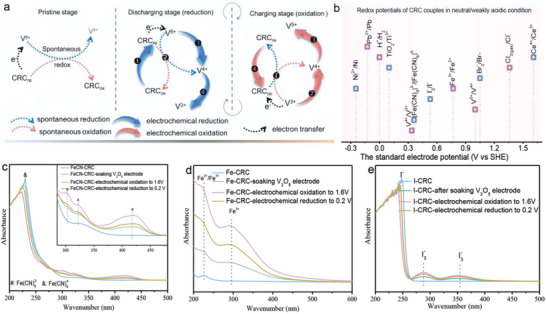
a) Schematic diagram for working mechanism of CRC in V^5+^/V^4+^ and V^4+^/V^3+^ redox reactions. b) The standard electrode potential of some redox couples in neutral/weakly acidic condition. UV–vis spectra of electrolytes with c) FeCN‐CRC, d) Fe‐CRC and e) I‐CRC at different stages.

Specific to the issues of V_2_O_5_, an extra CRC as “electron carrier” is introduced in ZnSO_4_ electrolyte to facilitate electron transfer in V^5+^/V^4+^ and V^4+^/V^3+^ redox reactions. As schematically illustrated in **Figure** **1**a for details, CRC_re_ which is capable of spontaneous electron transfer with V^5+^ is introduced in the V_2_O_5_//Zn battery to generate oxidized CRC (CRC_ox_) and more V^4+^ at the pristine stage. During the period of discharging process, the CRC_ox_ is electrochemically reduced into CRC_re_ and the spontaneous reaction between CRC_re_ and V^5+^ provides electrons for V^5+^/V^4+^ reduction, which is then electrochemically reduced to V^3+^. At the subsequent charging stage, CRC_ox_ spontaneously captures electrons from V^3+^ to form V^4+^ and CRC_re_ which is electrochemically oxidized to CRC_ox_ within the potential window of this system, and then it is on to the next cycle. Above all, CRC enables a positive contribution to enhance the electron transport by providing/capturing electrons in the reduction of V^5+^/V^4+^ and oxidation of V^4+^/V^3+^correspondingly, further promoting the electrochemical properties of V_2_O_5_//Zn battery. In light of the above design philosophy, the essential selection criteria of appropriate CRC can be summed up as follows: i) the reduced and/or oxidized CRCs can undergo a spontaneous chemical reaction with V_2_O_5_ electrode; ii) CRCs endow with the reversible electrochemical redox reaction within the potential window of V_2_O_5_//Zn battery system.

Based on the standard electrode potentials of some redox couples in neutral/weakly acidic condition (Figure 1b), the redox couples of [Fe(CN)_6_]^3−^/[Fe(CN)_6_]^4−^ (≈0.36 V), I_3_^−^/I^−^ (≈0.55 V) and Fe^3+^/Fe^2+^ (≈0.77 V, Fe‐CRC) are anticipated to be selected as CRCs. It is ascribed to that CRC_re_ can spontaneously provide and capture electrons to the reduction of V^5+^/V^4+^ (≈1.00 V) and the oxidation of V^4+^/V^3+^ (≈0.34 V), respectively.^[^ ^15^ ^]^ The electrochemical potentials of the mentioned CRCs are conducted by cyclic voltammetry (CV) of Zn‐based batteries with carbon paper cathodes in ZnSO_4_ electrolytes with 0.1 m K_4_Fe(CN)_6_, KI and FeSO_4_ additive, which are correspondingly denoted as CP//Zn in ZSFeCN, CP//Zn in ZSI and CP//Zn in ZSFe. Figure S1 (Supporting Information) shows reversible redox peaks at 1.2/0.9 V, 1.4/1.3 V and 1.5/1.3 V assigned to [Fe(CN)_6_]^3−^/[Fe(CN)_6_]^4−^, I_3_^−^/I^−^ and Fe^3+^/Fe^2+^ redox, demonstrating the selected CRCs own reversible electrochemical redox reactions within the operating voltage range of the V_2_O_5_//Zn battery. Furthermore, the changes in electrolyte composition of the assembled V_2_O_5_//Zn with CRCs at different stages are characterized by UV–vis spectroscopy. Taking FeCN‐CRC for example (Figure 1c), UV–vis spectrum of pristine K_4_Fe(CN)_6_ shows a major absorbance at ≈229 nm assigned to Fe(CN)_6_^4−^. After soaking V_2_O_5_ electrode in K_4_Fe(CN)_6_ solution, the Fe(CN)_6_^4−^ absorbance decreases accompanied by the increase of extra absorbance peaks at ∼302, ∼321, and ∼417 nm due to the [Fe(CN)_6_]^3−^, proving the spontaneous chemical reaction between [Fe(CN)_6_]^4−^ and V_2_O_5_ to form [Fe(CN)_6_]^3−^. The above phenomenon becomes more visible after electrochemical oxidation to 1.6 V, resulting from the electrochemical oxidation of [Fe(CN)_6_]^4−^ to [Fe(CN)_6_]^3−^, which is then electrochemically reduced to [Fe(CN)_6_]^4−^ after electrochemical reduction to 0.2 V. And the analogous results can be obtained in V_2_O_5_//Zn with Fe‐CRC and I‐CRC (Figure 1d,e). Integrating with the standard electrode potentials of CRCs and experimental results, [Fe(CN)_6_]^3−^/[Fe(CN)_6_]^4−^, I_3_^−^/I^−^ and Fe^3+^/Fe^2+^ redox couples are sufficient to meet the preliminary requirement of the CRCs for V_2_O_5_ electrode.

**Figure 2:**
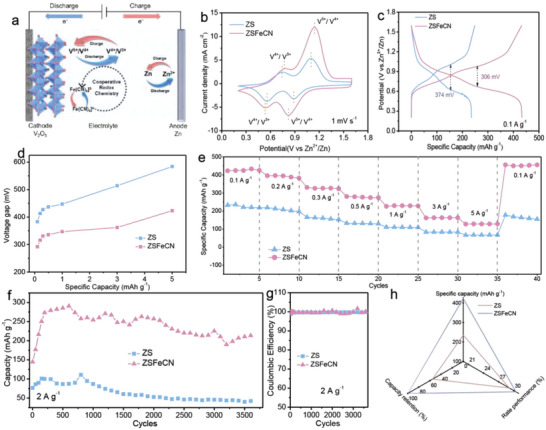
a) Schematic diagram of the V_2_O_5_//Zn batteries, b) CV curves at 1 mV s^−1^, c) GCD curves at 0.1 A g^−1^, d) Voltage gaps at various current densities, e) The rate performance, f) Cycling performance at 2 A g^−1^ and g) coulombic efficiency. h) The comparison of specific capacity, capacity retention after ≈3500 cycles and rate performance of ZS and ZSFeCN batteries.

To illustrate the positive role of CRC on the electrochemical performance of V_2_O_5_, commercial V_2_O_5_ without any pre‐treatment as cathode and the [Fe(CN)_6_]^3−^/[Fe(CN)_6_]^4−^ as CRC are first chosen for verification models. The X‐ray diffraction (XRD) pattern in Figure S2 (Supporting Information) is well assigned to the orthorhombic V_2_O_5_ (JCPDS#41‐1426), demonstrating the high purity of electrode material. The morphology of V_2_O_5_ is characterized with the scanning electron microscope (SEM), in which smooth nanosheets with a relatively large specific surface area are presented, potentially facilitating the electrolyte‐electrode interaction (Figure S3, Supporting Information). Besides the morphology, the homogeneous distribution of V and O elements for V_2_O_5_ sample are validated by the energy‐dispersive spectroscopy (EDS) in Figure S4 (Supporting Information). To further study the crystal phase and microstructure of V_2_O_5_, the transmission electron microscope (TEM) is performed and the nanosheet structure is observed in Figure S5a (Supporting Information), where the lattice fringes with a spacing of 0.296 nm is well‐matched with the (220) plane of V_2_O_5_ (Figure S5b, Supporting Information), in line with the SEM results and XRD pattern, respectively. The aqueous VZBs with V_2_O_5_ cathode, fiberglass diaphragm and Zn anode in 2 m ZnSO_4_ electrolyte without and with 0.1 m K_4_Fe(CN)_6_ (denoted as ZS and ZSFeCN, respectively) were assembled to exemplify the positive efficacy of CRC on electrochemical properties (**Figure** **2**a). As anticipated, CV curves of ZS and ZSFeCN batteries present characteristic redox peaks at around 1.1/0.8 V (V^5+^/V^4+^) and 0.7/0.5 V (V^4+^/V^3+^) without obvious peak shift at 1 mV s^−1^ between 0.2 and 1.6 V, suggesting the introduction of FeCN‐CRC shows no influence on the intrinsic redox reaction of V_2_O_5_ (Figure 2b). Comparatively, the higher current densities and larger CV curve area are observed in ZSFeCN by a large margin. Similarly, an improvement of specific capacity is observed in the galvanostatic charge‐discharge (GCD) curves from 232.6 mAh g^−1^ for ZS to 433.3 mAh g^−1^ for ZSFeCN at 0.1 A g^−1^, demonstrating the introduced FeCN‐CRC can obviously boost the capacity of the battery (Figure 2c). It is noteworthy that ZSFeCN shows a considerably lower voltage gap than that of ZS at series of current densities, implying that the existence of FeCN‐CRC enables a lower energy barrier and faster kinetics for redox reaction (Figure 2d). Also, we have studied the effect of different content of FeCN‐CRC additive on the electrochemical performance of V_2_O_5_//Zn battery. The capacity of V_2_O_5_//Zn battery was considerably boosted after adding FeCN‐CRC, and the battery with 0.1 m FeCN‐CRC additive exhibits the highest capacity (Figure S6, Supporting Information).

Subsequently, the rate performance of V_2_O_5_//Zn batteries are compared in Figure 2e. The ZSFeCN achieves conspicuous discharge specific capacities of 433.3, 395.7, 330.8, 280.6, 229.8, 163.0, and 128.6 mAh g^−1^ at various current densities from 0.1 to 5.0 A g^−1^, outperforming the ZS with capacities from 232.6 mAh g^−1^ to 67.8 mAh g^−1^. When the current density returns to 0.1 A g^−1^ after 35 cycles, the capacity of ZSFeCN restores to the initial state while that of ZS suffers from a certain extent of attenuation, indicating the enhanced cyclic stability of ZSFeCN. To further corroborate the superiority of FeCN‐CRC, the lifespan of V_2_O_5_//Zn batteries are investigated at 4.5 A g^−1^ via a consecutive GCD measurement. As depicted in Figure 2f, the ZSFeCN exhibits an admirable cyclability without obvious capacity degradation after approximately 3500 cycles (still remains 213.2 mAh g^−1^), in conjunction with ≈100% coulombic efficiency (CE, Figure 2g), in comparison, an inferior cycling stability with only 53.6% capacity retention (from 74.2 to 39.8 mAh g^−1^) is observed in ZS, demonstrating the crucial role of FeCN‐CRC in the stabilization of V_2_O_5_//Zn. The increased capacity of these two batteries during the first 250 cycles is mainly attributed to the electrochemical activation of the electrodes.^[^ ^14^, ^26^ ^]^ As for the capacity fluctuation in lifespan data might be ascribed to the temperature fluctuation (change at between 22 °C and 36 °C in our test days) during life test. All in all, the FeCN‐CRC enables ZSFeCN with superior electrochemical properties to ZS, including the capacity and lifespan (Figure 2h).

**Figure 3:**
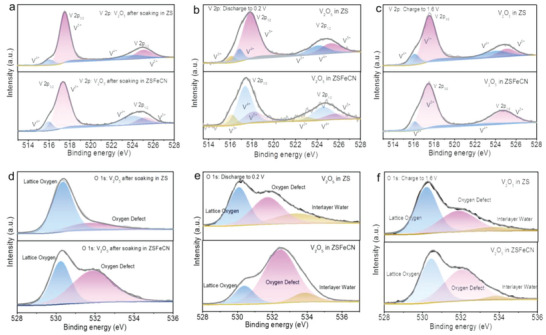
a) V 2p XPS spectra of V_2_O_5_ after contacting with ZS and ZSFeCN electrolytes; b) V 2p XPS spectra of V_2_O_5_ after discharging to 0.2 V in ZS and ZSFeCN; c) V 2p XPS spectra of V_2_O_5_ after charging to 1.6 V in ZS and ZSFeCN; d) O 1s XPS spectra of pristine V_2_O_5_ after contacting with ZS and ZSFeCN electrolytes; e) O 1s XPS spectra of V_2_O_5_ after discharging to 0.2 V in ZS and ZSFeCN; f) O 1s XPS spectra of V_2_O_5_ after charging to 1.6 V in ZS and ZSFeCN.

To further unravel the forenamed working mechanism of CRC in enhancing electrochemical performance of V_2_O_5_ electrode, semi‐in situ X‐ray photoelectron Spectroscopy (XPS) analyses for V_2_O_5_ cathodes of ZS and ZSFeCN batteries are collected. The V 2p XPS spectra of pristine V_2_O_5_ electrode after contacting with ZSFeCN electrolyte for 5 h in **Figure** **3**a shows slightly more V^4+^ content than that with ZS electrolyte, which attributes to spontaneous redox reaction of [Fe(CN)_6_]^4−^ and V^5+^ to form V^4+^. When discharging to 0.2 V, the V 2p XPS spectrum of V_2_O_5_ in ZSFeCN presents more V^4+^, V^3+^ and less V^5+^ compared to the V_2_O_5_ in ZS (Figure 3b). Subsequently, reduced V^4+^ and more V^5+^ content of V_2_O_5_ in ZSFeCN and [Fe(CN)_6_]^3−^ are demonstrated in the V 2p of V_2_O_5_ after fully charging to 1.6 V (Figure 3c). The above results present that FeCN‐CRC is competent for facilitating the reversibility of V^5+^/V^4+^ and V^4+^/V^3+^ redox reactions, further improving the electrochemical properties of V_2_O_5_//Zn battery. With regard to the O 1s XPS spectra of pristine V_2_O_5_ electrode after contacting with ZS and ZSFeCN correspondingly, two characteristic peaks at 530.3 and 531.7 eV representing the lattice and oxygen vacancy could be observed in Figure 3d.^[^ ^16^ ^]^ It shows that the oxygen vacancy forms in situ after contact with ZSFeCN due to the introduction of [Fe(CN)_6_]^4−^, which not only increases Zn^2+^ storage sites and ion‐diffusion paths, but also weakens the interaction between Zn^2+^ and the V_2_O_5_ host material, resulting in boosted capacity and cycling durability of V_2_O_5_.^[^ ^17^ ^]^ When discharging to 0.2 V, the peaks of interlayer water are detected in O 1s XPS spectra of V_2_O_5_ in both ZS and ZSFeCN, attributing to the hydrated Zn^2+^ insertion (Figure 3e).^[^ ^18^ ^]^ And a larger proportion of oxygen vacancies for V_2_O_5_ in ZSFeCN is also observed than that in ZS after discharging process. Moreover, a proportion of oxygen vacancy of V_2_O_5_ in ZSFeCN nearly restores to the original state after charging to 1.6 V, while that in ZS is not attainable, reflecting that the FeCN‐CRC enables the V_2_O_5_//Zn with enhanced redox reversibility (Figure 3f). These results bring into correspondence with the aforementioned design philosophy and conclusion. To explore the capacity contribution from the increased oxygen vacancies in V_2_O_5_ provoked in situ by CRC, the V_2_O_5_ electrode was pre‐soaked in mixed solution of 2 m ZnSO_4_ and 0.1 m K_4_Fe(CN)_6_ for 24 h, which was subsequently employed to assemble V_2_O_5_//Zn battery in 2 m ZnSO_4_ electrolyte (ZS‐24 h). As exhibited in Figure S7 (Supporting Information), the capacities of ZS‐24 h and ZSFeCN are 1.2 and 2.0 times as many as that of ZS correspondingly, which demonstrate secondary contribution of the increased oxygen vacancies in V_2_O_5_ provoked in situ by CRC on the improved capacity.

**Figure 4:**
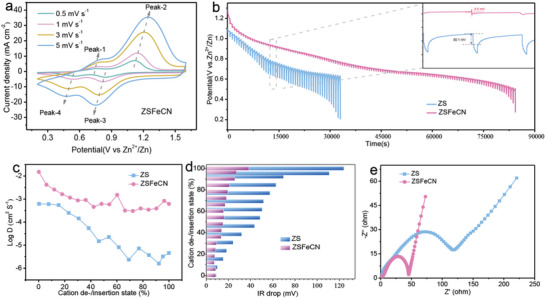
a) CV curves of ZSFeCN at various sweep rates; b) GITT curves at 0.15 A g^−1^, c) the Zn^2+^ diffusion coefficient and d) IR drop of ZS and ZSFeCN; e) Nyquist plots of ZS and ZSFeCN.

In an effort to account for the positive effect of FeCN‐CRC on enhanced electrochemical performance of V_2_O_5_, the interaction between electrolytes and electrodes are studied by comparing the contact angles on V_2_O_5_ electrodes in different electrolytes was first performed in Figure S8 (Supporting Information), in which similar contact angle in ZS (101°) and ZSFeCN (95°) electrolyte signifies the slight influence of the introduction of FeCN‐CRC on electrode‐electrolyte interaction. The multidimensional explorations for Zn^2+^ storage behavior are further carried out, mainly including the redox pseudo‐capacitance‐like contribution and the galvanostatic intermittent titration technique (GITT). First of all, the CV curves of V_2_O_5_//Zn battery at sweep rates from 0.5 to 5 mV s^−1^ are revealed in Figure S9 (Supporting Information) and **Figure** **4**a, in which the two pairs of cathodic and anodic peaks represent the multi‐step Zn^2+^ intercalation/de‐intercalation process. As previously reported, the electrochemical behaviors can be analyzed by the correlation of CV response current (*i*) and scan rates (*v*), which is based on the power‐law formula (Equation S4, Supporting Information).^[^ ^19^ ^]^ Thereinto, the value of *a* and *b* are adjustable parameters in Equation S4 (Supporting Information). When the value of *b* is approximately equal to 0.5, the capacity of batteries is contributed by the diffusion process, whereas the *b* value close to 1.0 indicates a capacitance‐controlled process. In view of this, the *b* values of ZS (0.7–1.0) and ZSFeCN (0.6–0.8) suggest that the capacities of both batteries are jointly controlled by the capacitive and diffusion characteristics (Figure S10, Supporting Information). Particularly, the relatively smaller *b* value of ZSFeCN signifies an enhanced proportion of diffusion contribution (Figure S11, Supporting Information), suggesting that FeCN‐CRC and in situ increased oxygen vacancies endow the V_2_O_5_ with faster kinetics of Zn^2+^ diffusion, which is advantageous to improve the Zn^2+^ storage capacity without the loss of high‐rate performance. Meanwhile, the percentages of capacitance and diffusion contributions at different sweep rates are obtained with Equation S5 (Supporting Information), in which *k_1_* and *k_2_* correspond separately to the proportionality coefficients of capacitive and diffusion‐controlled contribution.^[^ ^20^ ^]^ As shown in Figure S12 (Supporting Information), the ratios of capacitive contribution of 59.3%, 68.7%, 75.9%, and 83.4% (red shadow area) in ZSFeCN are slightly lower than those of ZS from 77.2% to 91.4% with the scan rates increasing from 0.5 to 5 mV s^−1^ (Figure S13, Supporting Information). Such a result is good agree with the aforementioned conclusion.

To gain an insight into the Zn^2+^ diffusion process in V_2_O_5_//Zn batteries, the GITT is performed at a current density of 0.15 A g^−1^. As depicted in Figure 4b, the obviously slower potential drop of ZSFeCN than ZS reflects the lower Zn^2+^ diffusion resistance, which can be attributed to the increase oxygen vacancy and the presence of [Fe(CN)_6_]^3−^/[Fe(CN)_6_]^4−^ redox couple. Quantitatively, the ion diffusion coefficients (IDC) of Zn^2+^ in ZSFeCN identified by GITT measurement is calculated to be 10^−1.8^–10^−3.5^ cm^−2^ s^−1^, superior to that of ZS with 10^−3.2^–10^−5.8^ cm^−2^ s^−1^, indicating a faster kinetics of V_2_O_5_ cathode in ZSFeCN (Figure 4c).^[^ ^21^ ^]^ Specifically, ZSFeCN displays a nearly ten‐fold reduction in voltage hysteresis than that of ZS during the whole discharging process in inset of Figure 4b,d, again proving the positive effect of FeCN‐CRC on the diffusion kinetics. Subsequently, the evaluation of battery kinetics is conducted by electrochemical impedance spectroscopy (EIS) measurement in Figure 4e. Compared to ZS, the Nyquist plot of ZSFeCN exhibits a smaller semicircular diameter at the high‐frequency region and a larger slope at low‐frequency region, suggesting an increased ion conductivity and diffusion rate of ZSFeCN, which account for the higher IDC of Zn^2+^ and reduced voltage hysteresis.^[^ ^22^ ^]^ Moreover, the electrochemical active area of electrodes is estimated with the correlation between the current densities and sweep rates at 0.02 V (vs saturated calomel electrode) in Figure S14 (Supporting Information). Noticeably, the ZSFeCN exhibits a larger slope than the ZS, implying increase available active sites for electrochemical reaction.^[^ ^23^ ^]^ On account of the abovementioned results, mechanism analyses and performance investigations have substantiated the positive effects of FeCN‐CRC on the enhanced electrochemical properties of V_2_O_5_ electrode, specifically demonstrated as follows: i) Spontaneous chemical reactions of FeCN‐CRC and V_2_O_5_ facilitate the electron transfer by capturing/providing electrons; ii) the increased oxygen vacancy in V_2_O_5_ not only provides more diffusion paths and active sites, but also reduces the interaction between Zn^2+^ and the V_2_O_5_ host material, which facilitate Zn^2+^ migration and storage as well as kinetics, thus improving the capacity and cycling durability of V_2_O_5_; iii) FeCN‐CRC endowing V_2_O_5_ with high ionic conductivity and ion diffusion rate can promote the Zn^2+^ storage capability.

**Figure 5:**
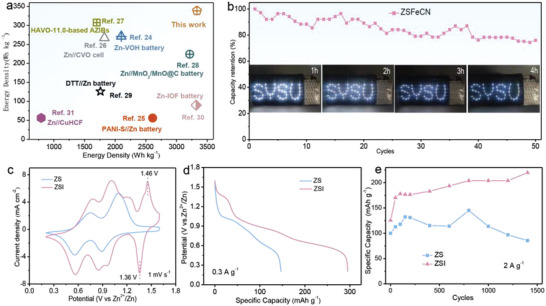
a) Ragone plots of the ZSFeCN and a few rechargeable Zn‐ion batteries;^[^ ^24^, ^25^, ^26^, ^27^, ^28^, ^29^, ^30^, ^31^ ^]^ b) Cycling performance of ZSFeCN pouch cell at ≈0.8 A g^−1^, inset is photographs of ZSFeCN pouch cells poweringand an LED device. c) CV curves at 1 mV s^−1^ and d) GCD curves at 0.3 A g^−1^; e) Cycling performance at 2 A g^−1^ of ZS and ZSI.

Also, the superiority of aqueous ZSFeCN is highlighted via the comparison of energy and power densities with those of previously reported aqueous energy storage devices. As illustrated in the Ragone plots in **Figure** **5**a, the ZSFeCN exhibits a maximum energy density of 338.6 Wh kg^−1^ and a peak power density of 3332.9 W kg^−1^, considerably exceeding most previously reported rechargeable Zn‐ion batteries, such as Zn‐VOH battery (271 Wh kg^−1^, 2100 W kg^−1^),^[^ ^24^ ^]^ PANI‐S//Zn (56 Wh kg^−1^, 2611 W kg^−1^),^[^ ^25^ ^]^ Zn//CVO cell (267 Wh kg^−1^, 1825 W kg^−1^),^[^ ^26^ ^]^ HAVO‐11.0‐based AZIBs (307.4 Wh kg^−1^, 1700 W kg^−1^),^[^ ^27^ ^]^ Zn//MnO_2_/MnO@C battery (224 Wh kg^−1^, 3215 W kg^−1^),^[^ ^28^ ^]^ DTT//Zn battery (126 Wh kg^−1^, 1760.8 W kg^−1^),^[^ ^29^ ^]^ Zn‐IOF battery (90 Wh kg^−1^, 3320 W kg^−1^)^[^ ^30^ ^]^ and Zn//CuHCF (56.3 Wh kg^−1^, 794.3 W kg^−1^).^[^ ^31^ ^]^ Moreover, the overall performances of ZSFeCN battery including the specific capacity, cyclic number, capacity retention, energy density and power density are superior to those of many reported VZBs in Figure S15 (Supporting Information), further corroborating the positive effect of FeCN‐CRC on the V_2_O_5_ electrode.^[^ ^17^, ^32^ ^]^ Beyond that, large‐scale ZSFeCN pouch cells with an area of 20 cm^−2^ were fabricated to exemplify the practical application feasibility of this strategy. As exhibited in Figure 5b, the ZSFeCN pouch cell appears an admirable durability with only 0.0048% capacity attenuation of each circle in 50 cycles averagely. Additionally, such a ZSFeCN pouch cell and a device comprised with three ZSFeCN were applied to operate an electronic timer and red LED bulb severally, which can work more than 4 h after only charging 2265 s at ≈0.4 A g^−1^ (inset of Figure 5b; Figure S16, Supporting Information).

To further verify the feasibility of as proposed strategy, the electrochemical performance of eligible I‐CRC and Fe‐CRC for V_2_O_5_//Zn (denoted as ZSI and ZSFe) are also investigated. As is visible in Figure 5c, the CV curve of ZSI battery displays an extra redox peaks at 1.46/1.36 V corresponding to I_3_^−^/I^−^ except for the two characteristic V^5+^/V^4+^ and V^4+^/V^3+^ redox peaks with larger CV curve area and higher response current densities compared to that of ZS. Additionally, the GCD curves of ZS and ZSI at 0.3 A g^−1^ exhibit that the I‐CRC enable the capacities of V_2_O_5_//Zn batteries to increase to ≈1.8 times (from 165.7 to 295.4 mAh g^−1^) (Figure 5d). When the current density increases to 5 A g^−1^, ZSI still shows the specific capacity of 179.7 mAh g^−1^, obviously superior to the ZS (67.8 mAh g^−1^), indicating the great rate property of ZSI (Figure S17, Supporting Information). And ZSI exhibits lifespan without any capacity fading after 1400 cycles, whereas the ZS shows 85.3% capacity retention after the same number of cycles in Figure 5e, confirming the positive role of CRC for V_2_O_5_ electrode. However, the lower capacity of ZSFe compared to the ZS was revealed in CV and GCD curves (Figure S18, Supporting Information), which is imputed to the side reaction between Fe‐CRC and Zn anode due to the high activity of Fe^2+^ (Fe^2+^ + Zn = Fe + Zn^2+^). The use of anion‐exchange membrane in ZSFe can effectively prevent their diffusion from anode side, which is highly hopeful to solve the adverse effects on Zn anode. Therefore, the suitable CRCs should be required to avoid any side reactions except for the selection criteria of chemical and electrochemical reactions mentioned above.

## 3 Conclusion

Summarily, we proposed and demonstrated the positive contribution of CRC as “electron carrier” on enhancing the eletrochemical properties of V_2_O_5_ cathode material for V_2_O_5_//Zn batteries. The appropriate CRCs are capable of facilitating the electron transfer by capturing/providing electrons for the redox of V_2_O_5_, in conjunction with increasing the ionic conductivity and ion diffusion rate to promote the Zn^2+^ storage capability. Additionally, the in situ increased oxygen vacancy in V_2_O_5_ can not only offer more Zn‐ion storage sites and diffusion paths, but also reduce the interaction between Zn^2+^ and the V_2_O_5_ host material to maintain the structural integrity, further improving the capacity and cycling durability of V_2_O_5_. Owing to these distinct merits, the ZSFeCN exhibits an approximately twofold increase of maximum specific capacity up to 433.3 mAh g^−1^ at 0.1 mA g^−1^, higher energy density of 338.6 Wh kg^−1^ and power density of 3332.9 W kg^−1^ compared with ZS battery. Additionally, a prolonged lifespan of ≈3500 cycles without capacity degradation is also achieved together with ≈100% CE. The reliable strategy together with the underlying mechanism and selection criteria of CRC in vanadium oxide cathode in this work provide novel insights into the design and production of reversible and stable cathodes for redox‐related batteries.

## Conflict of Interest

The authors declare no conflict of interest.

## Supporting information

Supporting Information

## Data Availability

The data that support the findings of this study are available from the corresponding author upon reasonable request.
