# Supplementary material for: Unraveling the Mechanism of Cooperative Redox Chemistry in High‐Efficient Zn2+ Storage of Vanadium Oxide Cathode
Source: Adv Sci (Weinh). 2023 Nov 14;11(1):2305749. doi: 10.1002/advs.202305749 (PMC10767404; doi:10.1002/advs.202305749)
Supplement: Supplementary file 1 — Supporting Information [file ADVS-11-2305749-s001.pdf]

ADVANCED SCIENCE
Open Access

Supporting Information

for *Adv. Sci.*, DOI 10.1002/advs.202305749

Unraveling the Mechanism of Cooperative Redox Chemistry in High-Efficient Zn^{2+} Storage of Vanadium Oxide Cathode

Lijun Zhou, Ping Li, Chenghui Zeng, Ang Yi, Jinhao Xie, Fuxin Wang, Dezhou Zheng*, Qi Liu and Xihong Lu**

Supporting Information

Unraveling the Mechanism of Cooperative Redox Chemistry in High-Efficient Zn^{2+} Storage of Vanadium Oxide Cathode

Lijun Zhou, Ping Li, Chenghui Zeng, Ang Yi, Jinhao Xie, Fuxin Wang, Dezhou Zheng,* Qi Liu, and Xihong Lu**

Experimental Procedures

Materials: The used chemicals and materials were of analytical grade, which can be utilized directly without any purification. Zinc sulfate heptahydrate ($ZnSO_4·7H_2O$), Potassium ferrocyanide ($K_4Fe(CN)_6·3H_2O$), Ferrisulphas ($FeSO_4$), Potassium iodide (KI), Vanadium pentoxide (V_2O_5), Hydrochloric acid (HCl, 37%), Ethanol absolute (C_2H_6O), Zn foil (thickness of 0.2 mm) and Carbon paper (thickness of 0.19 ± 0.01 mm) purchased from XinYe Electronic Material Factory and Shanghai Hesen Electric Co., Ltd, correspondingly.

Preparation of V_2O_5 cathode: To prepare the V_2O_5 electrode, the polyvinylidene fluoride (PVDF) was completely dissolved in 1-methyl-2-pyrrolidinone (NMP) solvent firstly. And then, the grinded mixed powder comprising commercial V_2O_5 powder and acetylene black was added into the above solvent, followed by an agitation at room temperature for 4 hours. Subsequently, the formed slurry was uniformly coated on the carbon paper and dried at 60 °C overnight to obtain the V_2O_5 electrode. As additional information, the weight ratio of V_2O_5 powder, acetylene black and PVDF was 8:1:1, and the mass loading of V_2O_5 electrode was 3.3 mg cm^{-2}, acquired by electronic scales (BT25S, 0.01 mg).

Preparation of ZS, ZSFeCN, ZSFe and ZSI electrolytes: 0.1 M $K_4Fe(CN)_6·3H_2O$ was dissolved in deionized water with magnetic stirring, followed by addition of 2 M $ZnSO_4·7H_2O$ and agitation at room temperature until homogenized, which was denoted as ZSFeCN. And the ZSFe and ZSI electrolytes correspondingly with 0.1 M $FeSO_4$ and 0.1 M

KI are obtained in the same way. As a comparison, 2 M $ZnSO_4 \cdot 7H_2O$ aqueous electrolyte was prepared without additives, denoted as ZS.

Assembly of vanadium-based Zn-ion cells: The aqueous ZS, ZSFeCN, ZSI and ZSFe 2032-type coin cells were assembled with the V_2O_5 cathode, glass fiber separator, Zn anode (thickness of 0.2 mm) in bare $ZnSO_4$ electrolyte and $ZnSO_4$ with 0.1 M $K_4Fe(CN)_6$, KI and $FeSO_4$ additive, respectively, in which the area of cathode and anode were 1.0 cm^2. Moreover, ZSFeCN pouch cells were also assembled with V_2O_5 cathode (1.2 mg cm^{-2}) and Zn anode with the same area of 20 cm^{-2} were fabricated.

Characterizations and electrochemical measurements: The electrolyte composition was analyzed by UV-vis absorption (Lambda 950). The morphologies, microstructures and element distributions of V_2O_5 samples were characterized by field-emission Scanning Electron Microscope (FE-SEM; JSM-6330F), energy dispersive X-ray spectroscopy (EDS; JSM-6330F) and Transmission Electron Microscope (TEM) (FEI Tecnai G^2 F30). The crystal structure and chemical state of V_2O_5 sample were respectively analyzed by X-ray diffractometry (XRD; D8 ADVANCE) and X-ray Photoelectron Spectroscopy (XPS, ESCALab250, Thermo VG) corrected by referencing C 1s to 284.8 eV. The contact angle tests of electrodes in different electrolytes were conducted via the Dataphysics OCA15 optical contact angle system. The electrochemical measurements of Zn-ion cells, involving cyclic voltammetry (CV), galvanostatic charge/discharge (GCD) measurements, electrochemical impedance spectroscopy (EIS, 0.01 Hz to 100 kHz) and galvanostatic intermittent titration technique (GITT), were carried out in an electrochemical workstation (CHI 760D).

Computational Details: The specific capacity, energy density and power density of cells were calculated from the discharge curve of GCD measurements based on the following formulas:

$$C = \frac{\int_0^{\Delta t} I \times dt}{m} \qquad (1)$$

$$E = C \times \Delta V \qquad (2)$$

$$P = \frac{C \times \Delta V}{\Delta t} \qquad (3)$$

Where C (mAh g^{-1}), E (Wh kg^{-1}) and P (W kg^{-1}) are the specific capacity, specific energy density and specific power density of cells correspondingly, Δt (h) is the discharging time, I (mA) is the specific discharging current in GCD measurements and m (mg) is the mass loading of electrode.

$$i = a v^{b} \qquad (4)$$

$$\mathrm{i} = k_1 v + k_2 v^{1/2} \qquad (5)$$

Where the value of *a* and *b* are adjustable parameters, *i* is CV response current, *v* is scan rates, k_1 and k_2 are correspond separately to the proportionality coefficients of capacitive and diffusion-controlled contribution.

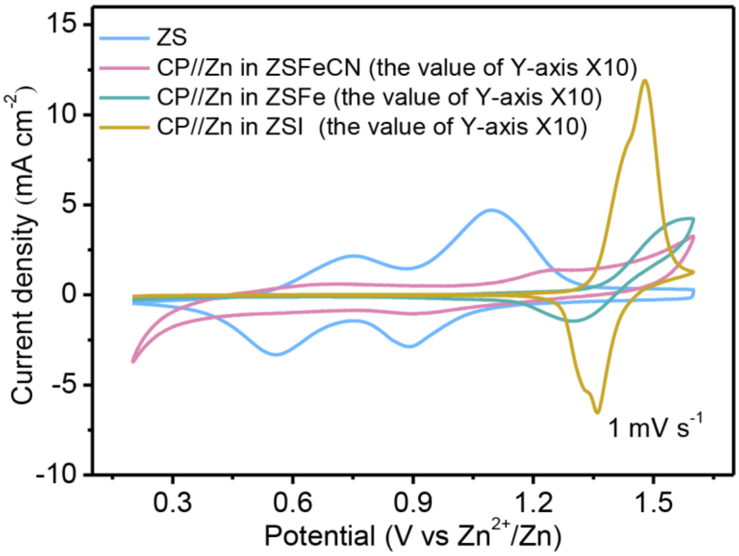

Figure S1. CV curves of ZS, CP//Zn in ZSFeCN, CP//Zn in ZSI and ZSFe at 1 mV s^{-1}.

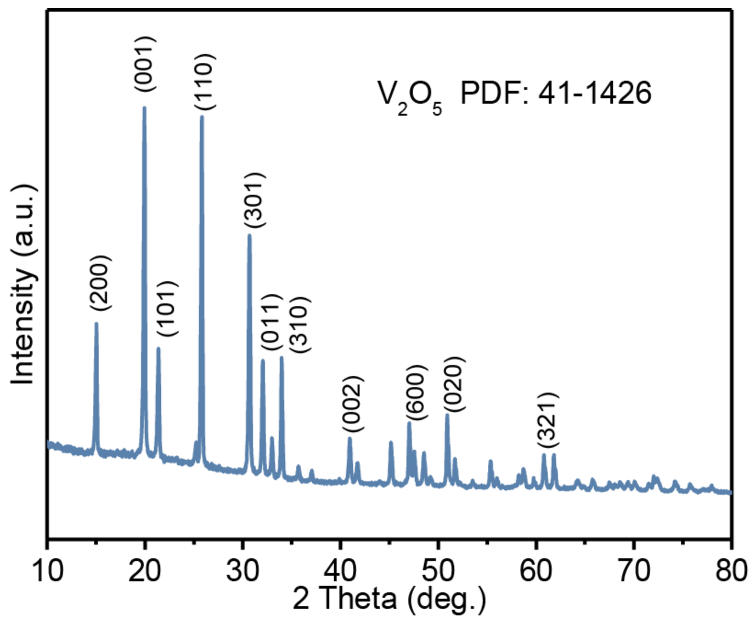

Figure S2. XRD pattern of the V_2O_5 sample.

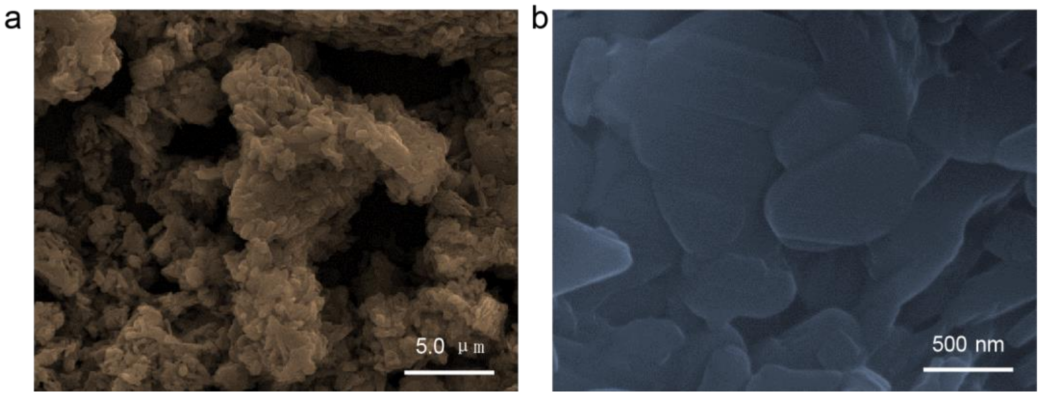

Figure S3. (a) SEM image, (b) high resolution SEM image of the V_2O_5 sample.

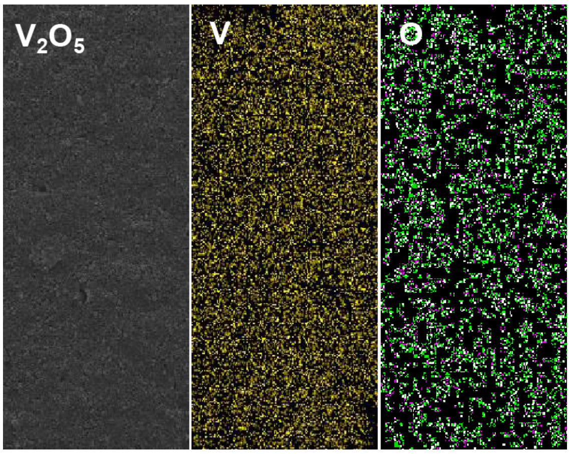

Figure S4. SEM selected-area elemental mapping images of V_2O_5 sample.

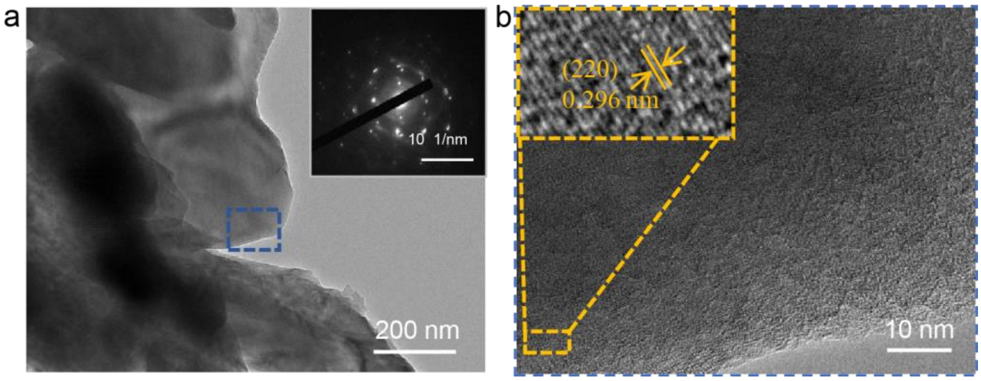

Figure S5. (a) TEM image (the corresponding SAED pattern is inset) and (b) high resolution TEM image from the blue outlined area inset (a) of the V_2O_5 sample.

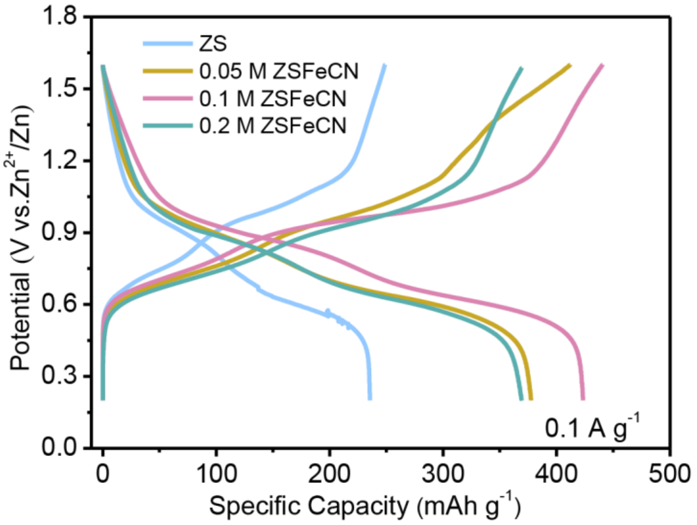

Figure S6. GCD curves of V_2O_5//Zn batteries with different content of FeCN-CRC additive at 0.1 A g^{-1}.

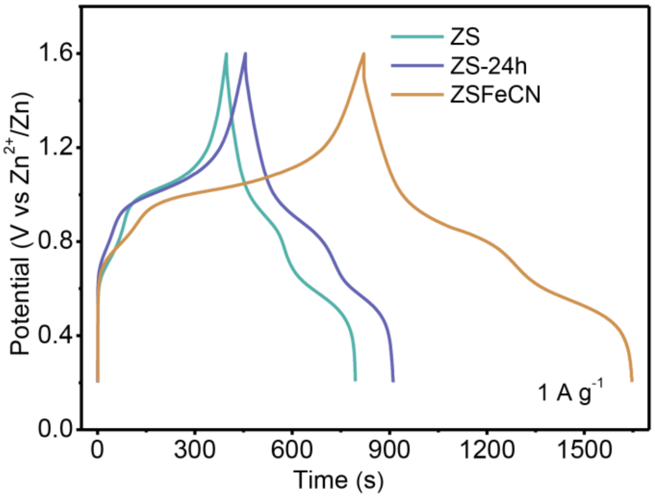

Figure S7. GCD curves of ZS, ZS-24h and ZSFeCN batteries at 1 A g^{-1}.

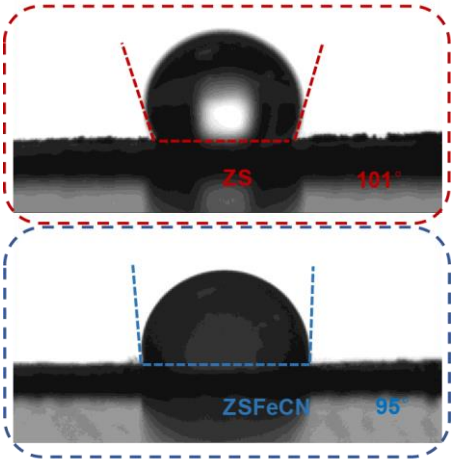

Figure S8. Water-based angle contact test of V_2O_5 in ZS and ZSFeCN electrolytes

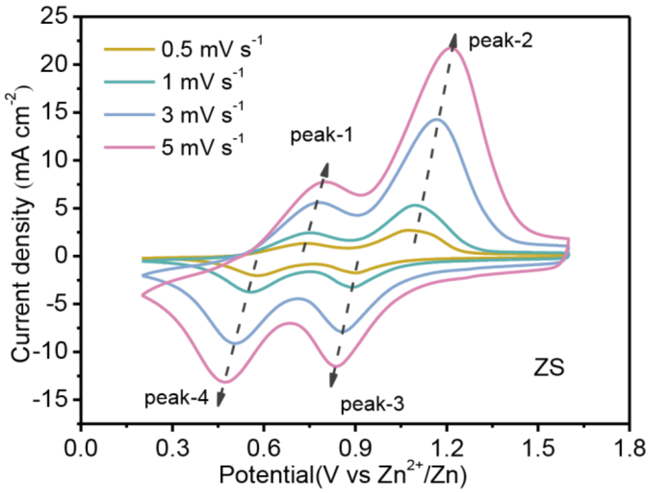

Figure S9. CV curves at various sweep rates of ZS.

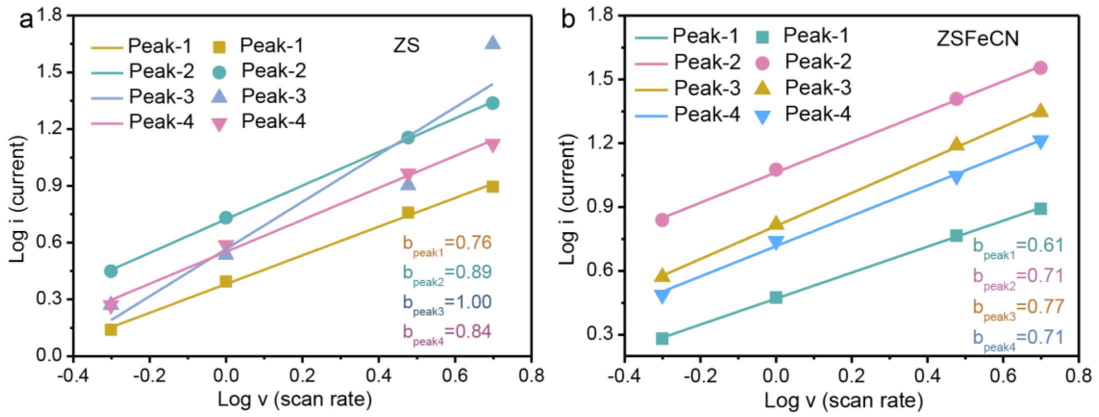

Figure S10. Log (peak current, mA cm^{-2}) versus log (scan rate, mV s^{-1}) plots and fitted slopes for the b values of (a) ZS and (b) ZSFeCN.

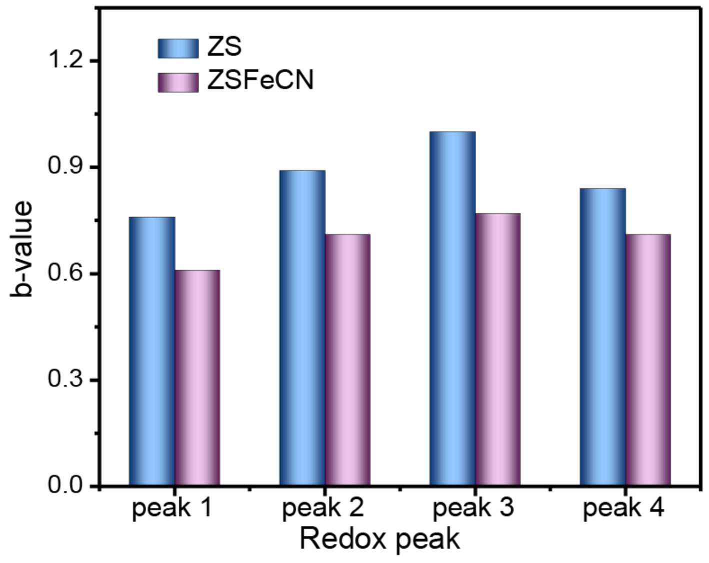

Figure S11. The comparison of b values between ZS and ZSFeCN.

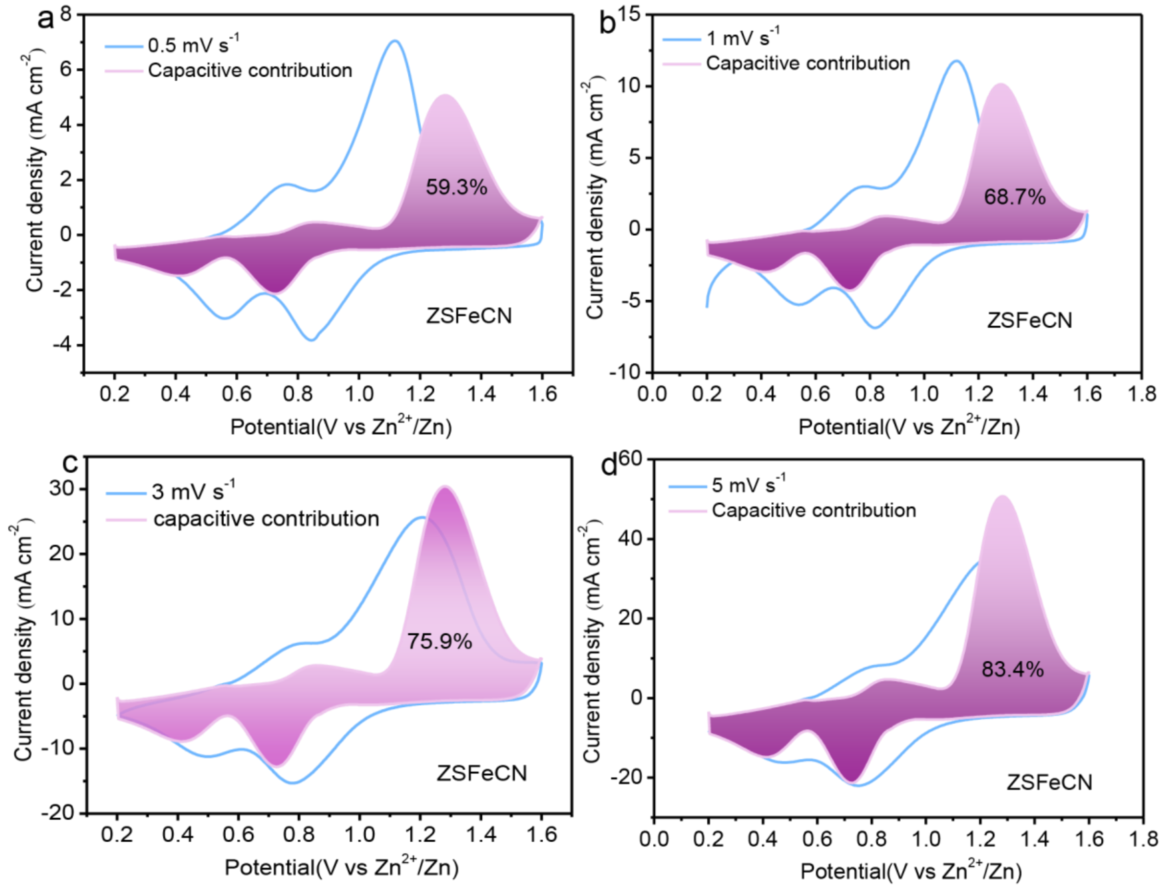

Figure S12. Capacitive contributions of ZSFeCN at (a) 0.5 mV s^{-1}, (b) 1 mV s^{-1}, (b) 3 mV s^{-1}, (c) 5 mV s^{-1}.

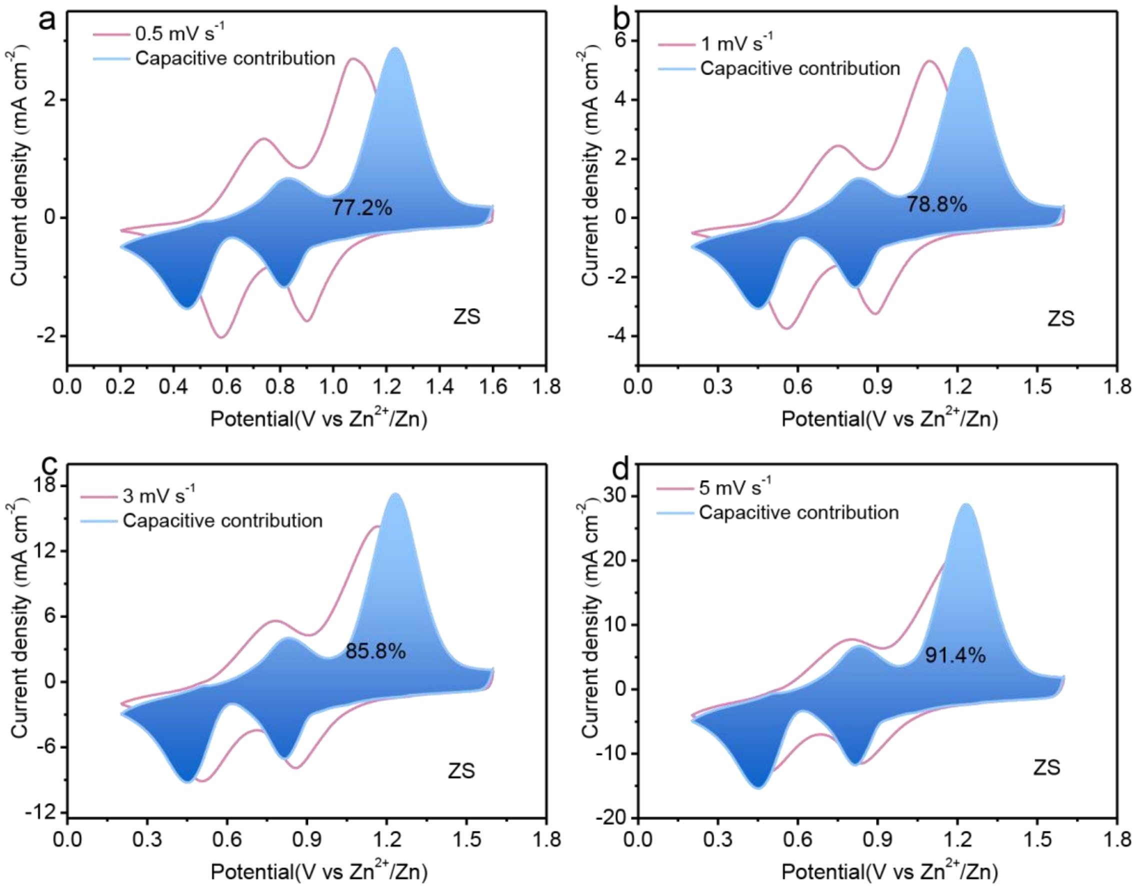

Figure S13. Capacitive contributions of ZS at (a) 0.5 mV s^{-1}, (b) 1 mV s^{-1}, (c) 3 mV s^{-1}, (d) 5 mV s^{-1}.

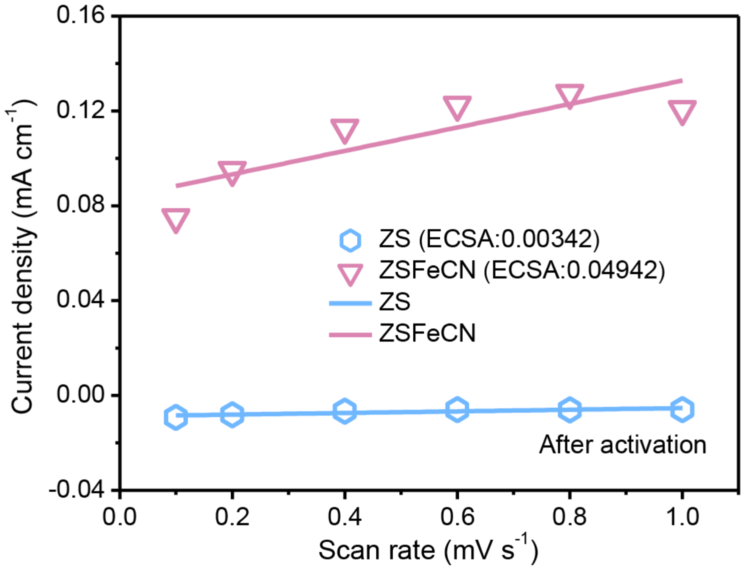

Figure S14. The relationship between current density and scanning rate at 0.02 V versus Hg_2Cl_2 of V_2O_5 in ZS and ZSFeCN electrolytes.

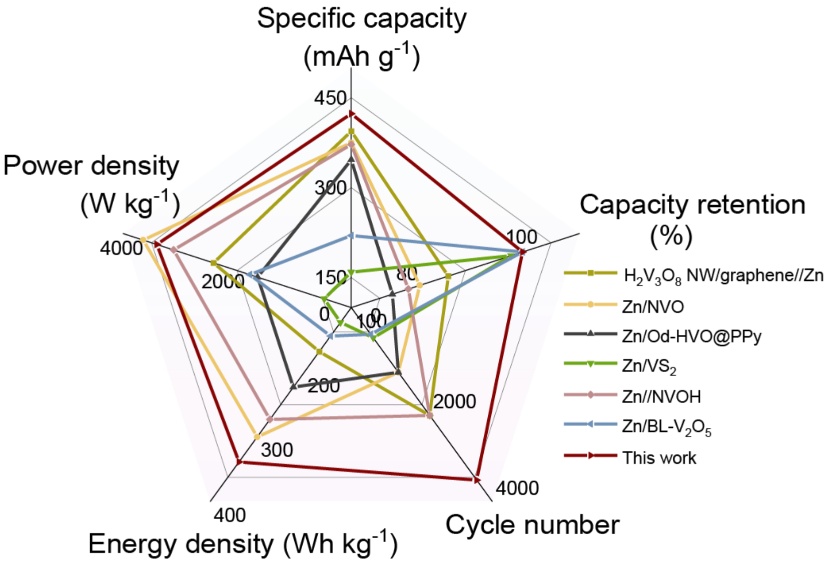

Figure S15. Comprehensive performance comparison of ZSFeCN with previously reported VZBs.

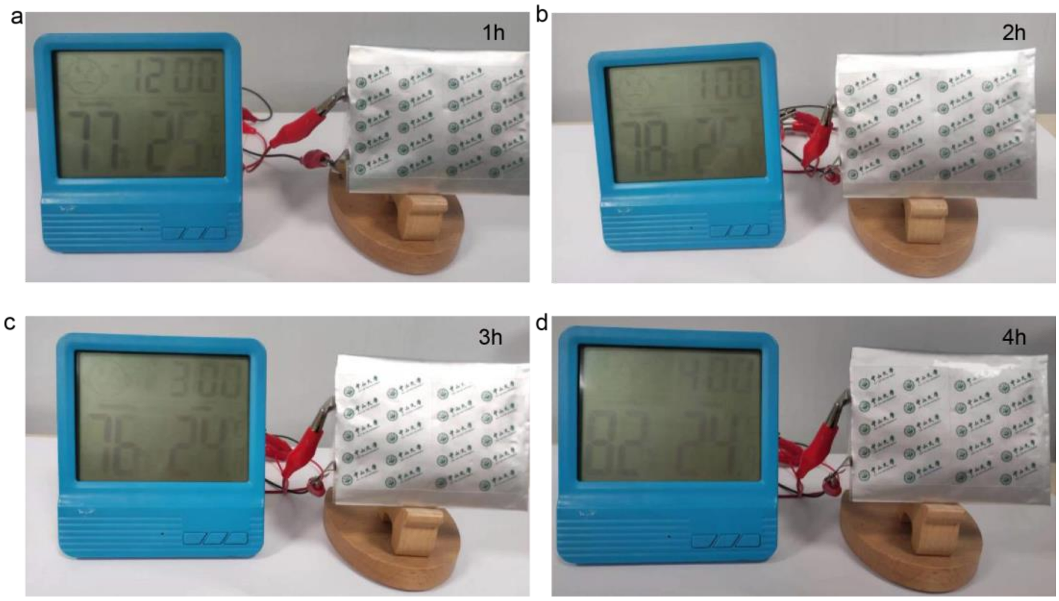

Figure S16. Photographs of ZSFeCN pouch cells powering and an electronic stopwatch.

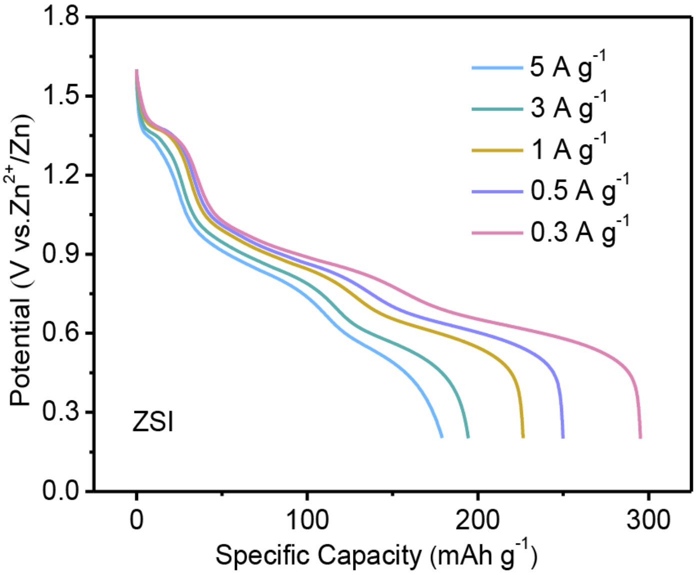

Figure S17. GCD curves of ZSI at various current densities.

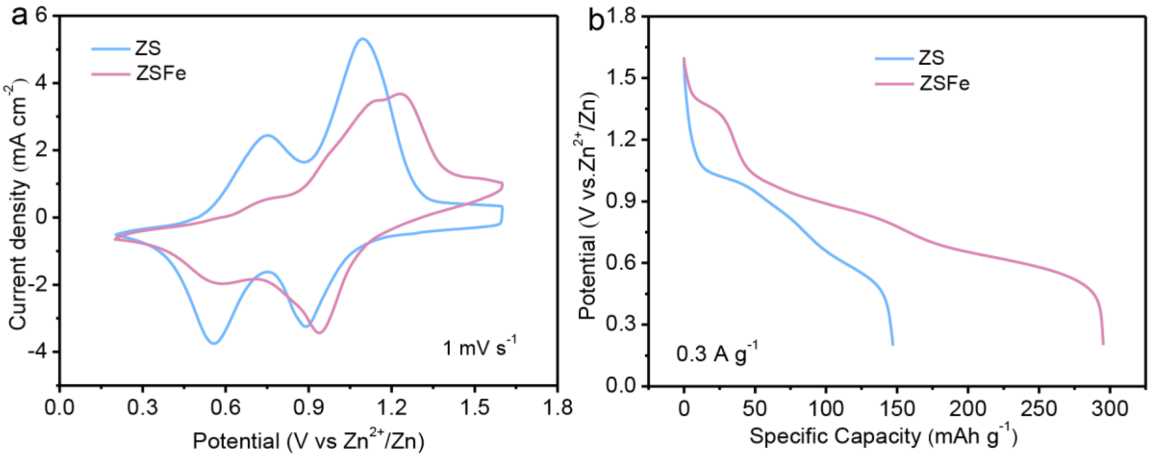

Figure S18. (a) CV curves at 1 mV s^{-1}, (c) GCD curves at 0.3 A g^{-1} of ZS and ZSF
